# Retroperitoneoscopic laparo-endoscopic single-site radical nephrectomy (RLESS-RN): initial experience with a homemade port

**DOI:** 10.1186/1477-7819-9-138

**Published:** 2011-10-28

**Authors:** Shiu-Dong Chung, Chao-Yuan Huang, Yao-Chou Tsai, Shih-Chieh Chueh, Shun-Fa Hung, Shuo-Meng Wang, Chun-Hou Liao, Hong-Jeng Yu

**Affiliations:** 1 Division of Urology, Department of Surgery, Far Eastern Memorial Hospital, Ban Ciao, Taipei, Taiwan; 2 Department of Urology. National Taiwan University Hospital, College of Medicine, National Taiwan University, Taipei, Taiwan; 3 Division of Urology, Department of Surgery, Buddhist Tzu Chi General Hospital, Taipei Branch, Taipei, Taiwan; Department of Urology, Tzu Chi University, Medical College, Hualien, Taiwan; 4 Cleveland Clinic, Glickman Urologic and Kidney Institute; Cleveland Clinic Lerner College of Medicine, Cleveland, OH 44195, USA; 5 Department of Urology, Cardinal Tien Hospital and College of Medicine, Ph.D. Program in Nutrition and Food Sciences, and Graduate Institute of Basic Medicine, Fu Jen Catholic University, Taipei, Taiwan

**Keywords:** Laparoendoscopic single-site surgery, LESS, nephrectomy, Retroperitoneum, renal cancer

## Abstract

We successfully performed 6 LESS radical nephrectomy via the retroperitoneal approach (RLESS) using the Alexis wound retractor as a single access with conventional laparoscopic instruments. The results demonstrated that our RLESS technique of radical nephrectomy is a safe and feasible procedure for management of localized renal cancer.

## Background

The novel technique laparoendoscopic single-site surgery (LESS) have been successfully performed in various urological operations that aim at performing laparoscopic surgery by consolidating all ports within a single skin incision, often concealed within the umbilicus and the transperitoneal route is typically employed [1-3]. The most obvious advantage of LESS is its cosmetic outcome when compared with conventional laparoscopic procedure [4]. Traditional laparoscopic techniques for radical nephrectomy usually need four to five trocars because retraction of intraabdominal organs is necessary [5-7]. There have been only limited reports of retroperitoneoscopic LESS procedures, and retroperitoneoscopic LESS nephrectomy was only reported very rarely, with limited case numbers, using variable LESS access platforms [8-10]. The present study retrospectively reviewed our experience of evaluating the feasibility and safety of retroperitoneoscopic LESS radical nephrectomy (RLESS-RN).

## Methods

Since June 2010, retroperitoneal LESS radical nephrectomy (RLESS-RN) has been performed in 6 patients. Perioperative data were collected retrospectively into our institutional review board-approved data registry and informed patient consent. All procedures were performed through the retroperitoneal approach.

### Operative technique

After the induction of general endotracheal anaesthesia, the patient was placed in a full flank position. The operating table was flexed at the waist level and the patient was securely fixed on the operating table with all pressure points well padded. Both the operator and the first assistant as the camera holder stood on the back side of the patient. All RLESS-RN was started from establishing retroperitoneoscopic working space by our previous reported method [11], with the open Hasson's technique and the modification that the space was dilated with the Preperitoneal Dissector Balloon (PDB 1000; Covidien, Mansfield, MA, USA) under the direct vision of a 0° 10-mm telescope instead of the original home-made dilatation balloon [12]. The skin incision was made over the mid-axillary line, half-way between the ipsilateral lower costal margin and the iliac crest. The retroperitoneoscopic working space was established, and the landmark of psoas muscle was identified. The original mid-axillary skin, muscular and fascial incision was then extended to 4 cm. The LESS platform also used an Alexis wound retractor (small, Applied Medical, Rancho Santa Margarita, CA). The Alexis wound retractor was placed in position through the incision with the bottom ring inside the retroperitoneal cavity. A pair of sterile surgical gloves was snapped onto the external ring, and the the gloves were ligated [13]. The first 10-mm port for the 30° degree telescope was inserted into the homemade access. The carbon dioxide insufflation for pneumoretroperitoneum was started up to the pressure of 15 mmHg, and we set up the second and third ports (one 5-mm port, and one 12-mm port). The ports were separated from each other as far as possible on the homemade single port. The basic principles and steps of the nephrectomy were similar to those of the conventional multiport retroperitoneoscopic nephrectomy by standard laparoscopic instruments including atraumatic bowel grasper, laparoscopic scissors, hook electrocautery, suction-irrigation, and Hem-o-lok clip [10,14].

However, the limitation by the space of retroperitoneal cavity, the instrument tip of the non-dominant hand needed to retract tissues some distance away from the point of dissection to facilitate the identification and dissection of the ureter and renal hilar vessels, which were ligated by Hem-o-lok clips and transected by laparoscopic scissors. Further dissection to mobilize the kidney will include all the Gerota's fat tissues. The adrenal glands were preserved in low pole cancer on preoperative imaging studies. After the kidney was totally detached from its surrounding tissues, it was extracted from the flank wound after the platform made by glove was removed, when its wound retractor was still in place to hold the wound as open as possible. Subsequently, the wound retractor was removed and the wound was closed in layers.

## Results

**Figure 1:**
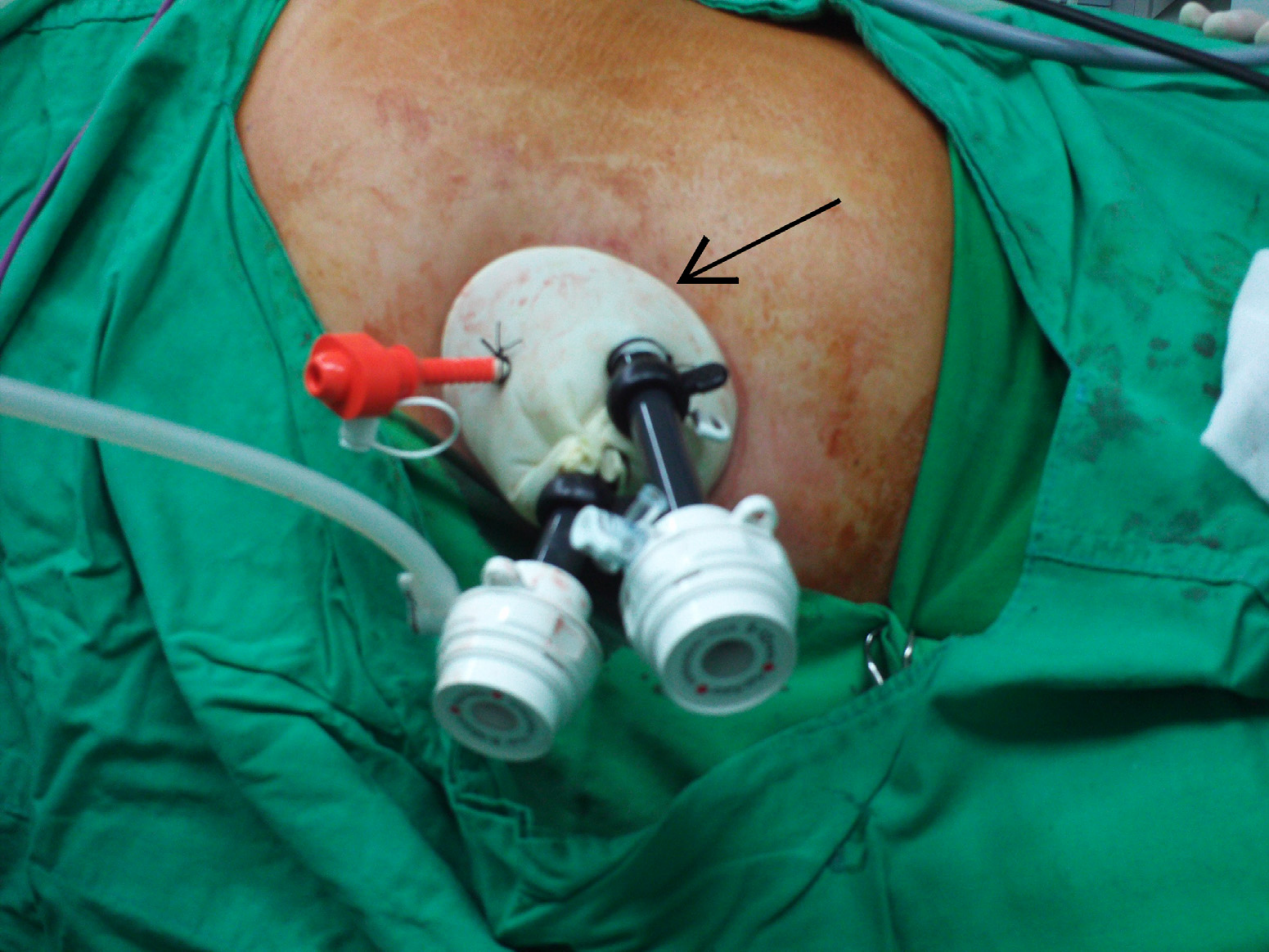
**The Alexis wound retractor was placed through the incision and three trocars (12-12-5 mm) were secured to the snapped double-layered latex surgical glove as the single port (homemade port) (arrow)**.

**Figure 2:**
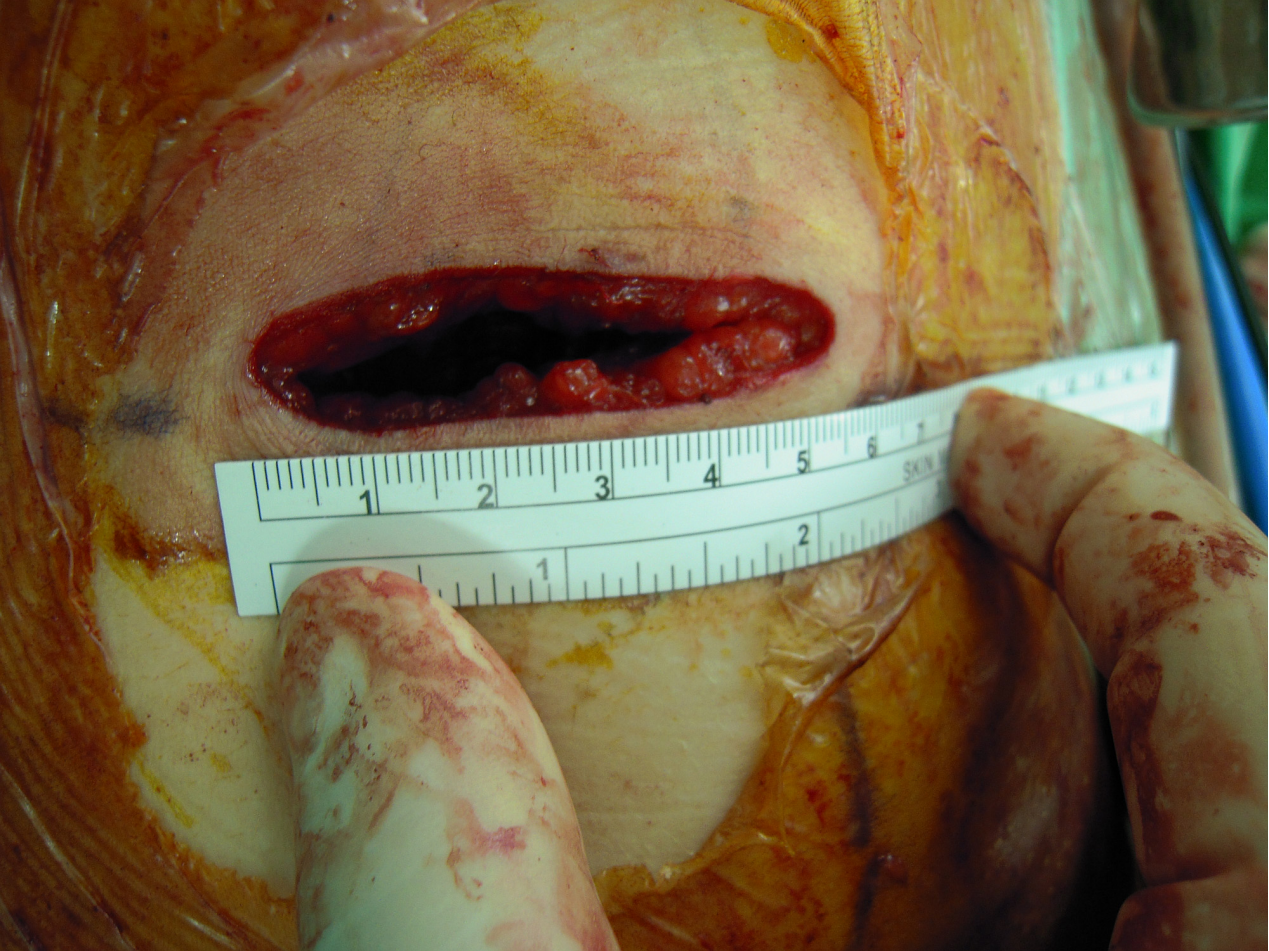
**Post-operative photograph following RLESS-RN and removal of kidney**.

The RLESS-RN was completed in all patients without conversion to the standard laparoscopy or open surgery. We performed the standard retroperitoneal laparoscopic surgical steps through our homemade single port (Figure 1). No additional ports or suspension sutures were applied in this present study. The demographic data of these patients are summarized in Table 1 and the perioperative data are summarized in Table 2. In the present series, the mean operative time and estimated blood loss were 235 minutes (range 190-335 minutes) and 42 mL (range 10-100 mL), respectively. All the specimens were retrieved from the flank wound successfully (Figure 2); no separate incision was required to remove the specimens. All the other procedures were performed through a single port exclusively with only traditional laparoscopic instruments. The mean time of oral intake after surgery was 45.4 hours days (range 12-72 hours), and the average duration of hospital stay after surgery was 5.8 days (range 5-8 days). No intraoperative or early major complication occurred. The pathology revealed 5 cases of renal cell carcinoma and one case of oncocytoma with a mean specimen weight of 217 gm (range 92-327 gm).

**Table 1:** Patient demographic data

N	Sex	Age	BW(kg)	Ht(m)	BMI	ASA	**P.H**.
1	M	78	47.2	1.51	20.7	III	Nil
2	F	58	59	1.63	22.2	III	Nil
3	F	55	51	1.59	20.2	III	ESRD
4	F	55	51.8	1.49	23.3	II	Nil
5	M	27	83.1	1.87	23.8	II	Nil
6	F	24	58.9	1.54	24.8	II	Nil

BW, body weight; Ht, height; BMI, body mass index; ASA, American Society of Anesthesiology;

P.H., past history; ESRD, end-stage renal disease

**Table 2:** Perioperative Data

N	OP time (min)	length (cm)	EBL (mL)	MSO4 (mg)	DOS (days)	Pathology report	Histology	Specimen wt (gram)	Kidney size (cm)
1	220	8	20	15	8	RCC	clear cell	197	12 × 5.5 × 4
2	335	7	100	5	5	RCC	clear cell	327	10 × 7 × 6.1
3	200	6	300	45	14	RCC	clear cell	92	8 × 5 × 3
4	190	5.5	50	35	6	Oncocytoma	-	165	10 × 4.5 × 4
5	190	7	30	40	5	RCC	clear cell	258	11 × 7 × 5
6	240	7	10	10	5	RCC	papillary	263	14 × 7 × 3.5

EBL, estimated blood loss; MSO4, Morphine Sulfate; DOS, days of stay

## Discussion

In our initial series reported in the present study, we have successfully shown that RLESS-RN using our homemade single port with standard laparoscopic instruments is feasible and safe, and have provided acceptable operative outcomes. In addition, the perioperative results in the present study were comparable to those of the pioneer LESS series [9,10,15-19]. Since the first laparoscopic nephrectomy performed by Clayman et al [20] in 1991, several reports have showed the advantages of laparoscopic or retroperitoneoscopic nephrectomy on decreased postoperative pain, shorter hospital stay, more rapid convalescence, and improved cosmesis compared with open surgery [6]. Long-term oncologic outcomes are comparable.^6 ^The evolution of urologic surgery is aimed at developing a scarless technique. The concept of LESS surgery is based on improved cosmetic results, faster return to work, and reduced occurrence rate of port-site hernia [21,22]. In contrast laparoscopic operation, potential disadvantages of the retroperitoneoscopic procedure include the inability to hide the wound in the umbilicus to make it 'scarless' compared to the transperitoneal LESS nephrectomy.

When compared with conventional retroperitoneoscopic nephrectomy, there is a steeper learning curve for surgeons, mainly as a result of clashing of instruments and lack of triangulation, which can be addressed in part by the use of articulating instruments. However, available laparoscopic articulating instruments can be difficult to use and generally nonergonomic [21]. To overcome the challenges, da Vinci Surgical System, which provide intuitive articulation with EndoWrist technology has been applied to LESS (R-LESS) using the GelPort as an access platform, this method provides adequate spacing and flexibility of port placement and acceptable access to the surgical field for the assistant [23]. Most series use an umbilical site of entry to the peritoneal cavity, of which the space allowed the robotic system establishment, the reports regarding retroperitoneal LESS assisted by robotis system remains scarce. The surgical team led by Kaouk recently reported their initial experience of robotic assisted LESS-RN (R-LESS RN) and demonstrated that perioperative outcomes comparable to conventional laparoscopic radical nephrectomy [24]. In addition, their R-LESS RN technique reduced analgesic use and a decreased hospital stay.

Multiple reports suggest a modest advantage for LESS when compared with standard laparoscopy in terms of convalescence and postoperative pain.^3, 18,21 ^In our experience with standard grasper in the non-dominant hand and a conventional laparoscopic instrument in the dominant hand, the retroperitoneoscopic LESS nephrectomy is still feasible though the access of our homemade single port setting. In contrast to our previous method applying in laparoscopic LESS, we preserved the finger parts of the glove to maintain adequate space to avoid too bulky instruments trafficking in the limited retroperitoneal cavity.

Compared with the transperitoneal LESS surgery, there are several obvious advantages to RLESS-RN. For instance, RLESS-RN provides easier access with conventional straight laparoscopic instruments; most notably, the distance and angle to dissect the upper pole of the kidney or the adrenal gland is much easier. As in our series, we do not have to insert any additional port and instrument to retract the liver, spleen or bowels away from the exposure. In addition, RLESS-RN has minimal risk to bowel injury during dissection. In patients who had previous major abdominal surgery or are morbid obesity and extremely much fat of the anterior abdomen, the retroperitoneoscopic LESS approach offers direct access to the kidneys without dissecting the bulky adipose tissue or violating the integrity of the peritoneal envelop.

Compared with conventional retroperitoneoscopic nephrectomy, RLESS-RN reduced patients' discomfort and potential complications related to the port wounds including hernia and wound infection. Ryu et al performed 2 radical nephrectomies by RLESS-RN, however, the specimen was retrieved by another lower abdominal Gibson incision. We directly removed the whole specimen through the original flank incision only with or without extension of the wound [9]. Based on their results, Ryu et al suggested that RLESS procedures provided subjective cosmetic benefit and could be a useful option in organ-ablative and nonextirpative surgery [9]. White et al. also reported eight successful cases of retroperitoneoscopic LESS surgery and suggested that this technique is feasible and offers comparable surgical outcomes and superior cosmesis and pain control compared to conventional retroperitoneoscopic surgery. However, no case of radical nephrectomy was reported in their series [19].

Several types of single ports are currently commercially available; however, these devices were not globally available, including in Taiwan. Hence, we created our homemade single port with multiple access channels by securing several standard laparoscopic ports onto this homemade port after having experienced the procedure [13]. The homemade single-port is more cost-effective than present single-port entry systems and highly flexible for its freedom in choice of trocar sizes and trocar position arrangement. In addition, the device is a durable even in procedures required long operative times. Although the case number is small, we presented the first series of RLESS-RN for renal cancer and demonstrated the removal of specimen without any other incision. RLESS-RN by using our homemade single port as an access platform is feasible and safe, and provides comparable preoperative outcomes.

## Conclusions

Our initial experience revealed that RLESS-RN is a safe and feasible procedure for renal cancer with improved cosmetic outcomes. Further prospective and long term studies are warranted to provide more powerful evidence with regard to peri-operative benefits and oncologic control.

## Competing interests

The authors declare that they have no competing interests.

## Authors' contributions

SDC and CYH drafted the manuscript. SMW and CYH carried out the operations. SDC, YCT and SFH participated in the design of the study and performed the statistical analysis. SCC and HJY conceived of the study, and participated in its design and coordination. All authors read and approved the final manuscript.
